# Editorial: Understanding Trajectories and Promoting Change From Early to Complex Skills in Typical and Atypical Development: A Cross-Population Approach

**DOI:** 10.3389/fpsyg.2021.647464

**Published:** 2021-02-19

**Authors:** Alessandra Sansavini, Klaus Libertus, Annalisa Guarini, Melissa E. Libertus, Mariagrazia Benassi, Jana M. Iverson

**Affiliations:** ^1^Department of Psychology, University of Bologna, Bologna, Italy; ^2^Department of Psychology, University of Pittsburgh, Pittsburgh, PA, United States; ^3^Learning Research and Development Center, University of Pittsburgh, Pittsburgh, PA, United States

**Keywords:** developmental trajectories and cascades, relations across developmental domains, caregiver-child interaction, early interventions, atypical development

Theoretical perspectives such as neuroconstructivism, dynamical systems, or developmental cascades (Thelen and Smith, 2007; Karmiloff-Smith, 2009; Iverson, 2010; Masten and Cicchetti, 2010) suggest that early perceptual, motor, cognitive, and communicative skills are related in development with cascading effects of one domain on multiple, seemingly unrelated domains. In particular, dynamical systems views emphasize that development is experience-dependent, characterized by a continuous interplay between the developing individual, their structural and functional constraints, and their social and physical environments. In recent years, several empirical studies have reported findings supporting these theories, showing that sensory and motor development may influence cognitive skills, language, social interaction, literacy development, numeracy, and academic achievement (e.g., D'Souza et al., 2017; Libertus and Hauf, 2017; Zuccarini et al., 2017). These findings suggest that early developmental risk factors may depend on the interplay among genetic, biological, and environmental factors (Sansavini et al., 2011; LeBarton and Iverson, 2016; Thomas et al., 2020). Taking such an integrative view of development raises several important questions. For example, early identification of children at risk, their developmental trajectories, possible sub-optimal outcomes, and effects on the environment as well as the role of protective factors or the effectiveness of various early interventions need to be more deeply investigated across different developmental domains and populations.

By referring to the above integrative view, the current Research Topic includes 21 articles covering theoretical and applied perspectives on three main issues: (i) cascading effects of early motor, perceptual, attention, communicative, language, and cognitive skills, (ii) impact of environmental factors (e.g., caregivers' behaviors, interventions, and cultural contexts) on development, and (iii) cross-domain relations. These issues are investigated across populations with typical, at risk or atypical development.

## Cascading Effects of Early Motor, Perceptual, Attention, Communicative, Language and Cognitive Skills

Several contributions examine the cascading effects of early motor, perceptual, communicative, language, and cognitive skills. Two articles provide an overview of this area by reviewing the existing literature. Gonzalez et al. review the predictive capacities of gross and fine motor skills for language outcomes in typical development from birth up to 5 years of age. The review highlights that changes in motor development provide children with new learning opportunities to interact with objects, their environment, and caregivers, with both gross and fine motor skills related to language outcomes. Similarly, Vissers et al. review the development of inner speech. They identify a fourth stage, i.e., condensed inner speech, and argue that inner speech impairment may account for cognitive deficits in children with developmental language disorder, hearing loss, and autism. Implications for assessing and stimulating inner speech during interventions in clinical populations are discussed.

In addition to these reviews, several empirical studies report new results on the cascading effects of development in one domain on other domains contributing to identifying early predictors of developmental delays. Zuccarini et al. examine the intra-domain and cross-domain cascading effects of early gross motor skills on later motor and cognitive development in infants born extremely preterm and full-term. Gross motor skills at 6 months of age relate to gross motor, fine motor, and cognitive skills at 12 months and predict gross motor delays. These findings highlight the importance of assessing gross motor, fine motor, and cognitive skills early, especially in extremely preterm children. Bettoni et al. find associations between the ability to learn and generalize abstract rules from sequences of visual shapes at 7 months of age and grammatical skills at 2 years of age, providing one of the first pieces of evidence that rule learning mechanisms are involved in language acquisition and can act as an early neurocognitive marker for language impairments. van Baar et al. examine early attention problems that can hinder cognitive and socio-emotional development. Their results show that lower attention skills at 18 months predict slower cognitive development at 24 months suggesting that assessments of early attention capacities can be useful predictors for development across domains. Luke et al. also examine the predictive value of early skills for later development by focusing on early communicative skills such as pointing and iconic gestures. Their results show that index-finger pointing at 12 months and comprehension of iconic gestures at 3 years of age predict language skills between 5 and 6 years of age in typically developing children and children with a language delay or developmental language disorder. Sansavini et al. focus on the role of deictic, iconic, and representational gesture production at 18 months in order to identify language delays between 18 and 36 months in children with genetic or biological risks and low-risk peers. Their results reveal that low rates of pointing at 18 months are a marker of language delay both in siblings of children with autism spectrum disorder and in extremely preterm children. The findings of both Luke et al. and Sansavini et al. support the assumption of an integrated speech–gesture communication system, with limited pointing production acting as an early marker of language delay.

Two further studies examined the relations between language and cognitive skills at preschools age and oral and written language skills at school age. Perez-Pereira et al. examine through a mediation model the relation among executive functions at age 4 and 5, language measures and phonological awareness at age 5, and reading abilities at age 9 in low-risk preterm children compared to full-term children. Their findings demonstrate that at age 9 single word reading is directly related to preschool syllabic awareness, whereas written text comprehension to preschool working memory and syntactic comprehension. Ebert bring new evidence of the relation between early language and socio-cognitive (i.e., theory of mind) skills at preschool age and higher-order metacognitive and language skills at school age up to early adolescence, highlighting through a mediation model, that oral text comprehension in early adolescence is related to earlier mental state language, language skills and theory of mind, whereas written text comprehension to same age oral text comprehension and earlier mental state language and metacognitive knowledge.

## Impact of Environmental Factors on Development

Several studies reported in this Research Topic examine the impact of environmental factors (e.g., caregivers' behaviors, interventions, and cultural contexts) on development. Provenzi et al. provide a review of evidence supporting the application of Video-Feedback Intervention with parents of children with neurodevelopmental disabilities (e.g., cerebral palsy, sensory and/or psychomotor delay, and genetic syndromes). The review, including 10 records from the late 80's up to 2020, suggests that the application of Video-Feedback Intervention is associated with better children communicative, cognitive and social outcomes, reduced behavioral problems and increased parental caregiving skills; however, it also raises several methodological questions highlighting the need of further evidence-based clinical practice.

Besides this review, several empirical studies report new results on the role of environmental factors on early development. Della Longa et al. show that 5-month-old infants, stroked with a brush at slow velocity, display a preference for a visual–tactile synchronous video. These findings suggest that affective touch might play a critical role in the early development of bodily self-awareness and the distinction between one's self and others with potential cascading effects on the development of interpersonal engagement and social cognition abilities. Neri et al. examine the impact of the severity of low birth weight, as well as of maternal anxiety at 3 months of infants' corrected age, on infants' outcomes during the first year postpartum. Their results show that the severity of low birth weight impacts on performance quotient contributing to lower scores with respect to full-term infants, as well as on hearing and language and locomotor scores in interaction with maternal anxiety (i.e., tendency to worry) contributing to decreasing scores during the 1st year of life. These findings suggest that interventions targeting parental functioning may be particularly effective for infants born preterm or with low birth-weight. Oudgenoeg-Paz et al. investigate cross-cultural differences in Dutch and Israeli parental practices and beliefs related to motor development in the 1st year of life. They note that Israeli parents practice infant prone positioning more, whereas Dutch infants spend substantially more time in the playpen, highlighting the cultural diversity of parental practices and their significant impact on infant motor developmental trajectories.

Three other studies examine the impact of environmental experiences on numerical development at preschool age. Bernabini et al. examine the role of home numeracy activities, parents' and children's cognitive and linguistic skills and Approximate Number System (ANS), i.e., the ability to estimate and compare numerical quantities without counting, in supporting early math skills in children in their last year of kindergarten. They show that home numeracy activities and children's ANS skills predict children's early math skills better than parent and other child variables. Similarly, Libertus et al. investigate the effect of a visual ANS training on math abilities, comparing it to a phonological awareness training. Children who completed visual ANS training show significant improvements in auditory ANS precision and math ability compared to children who completed phonological awareness training. These results provide evidence of an early modality-independent ANS that seems causally linked to math ability. Furthermore, Pellizzoni et al. found substantial differences in executive functions (i.e., working memory and inhibitory control) and early numerical abilities (i.e., counting, digit quantity mapping, and digit naming skills) between deprived groups of children, i.e., Yazidis and Syrian refugees attending psycho-social-support activities in their countries, and Italian children attending the 3rd year of kindergarten, highlighting the heavy impact of living in socio-economically disadvantaged and deprived contexts on cognitive development and the need of tailored interventions for these children.

## Cross-Domain Relations in Populations With Atypical Development

Lastly, some papers investigate cross-domain relations in populations with atypical development. Two studies examine emotional and cognitive processes in individuals with Down Syndrome and a matched typically developing group. Roch et al. analyze the recognition of basic emotional expressions from faces and words highlighting similar developmental trajectories in the two groups, as far as the processing of simple visual and linguistic stimuli conveying basic emotions is concerned. Mento et al. focus on the ability to implicitly build up subjective statistics of events' temporal structure in order to prepare for future actions. They find that individuals with Down Syndrome are not sensitive to global rule change, while a group of typically developing matched individuals is sensitive to global rule change suggesting that the use of flexible cognitive mechanisms to implicitly extract high order probabilistic rules to build-up internal models of event temporal properties is disrupted in individuals with Down Syndrome. Together, the findings from these two studies may contribute to better educational and psychological interventions targeting emotion and cognitive processes for individuals with Down Syndrome.

Three other studies investigate symptomatologies in neurodevelopmental or psychopathological disorders. Giovagnoli et al. investigate the relation between developmental dyslexia and internalizing symptomatology highlighting an increased level of self-perceived anxiety, depression and somatic symptoms in adolescents with respect to primary school children. In adolescents with dyslexia, high levels of internalizing symptoms are associated with low self-esteem and hyperactivation to problematic situations. By contrast, low symptom severity is associated with positive relationships with peers suggesting that remediation programs for dyslexia should include implementing motivation strategies, self-esteem enhancement activities, and building peer networks. Carta et al. investigate comorbidity between attention-deficit/hyperactivity disorder and autism spectrum disorder highlighting the role of externalizing problems in identifying a clinical intermediate phenotype. Indeed, youths with co-occurrence of these two disorders show externalizing problems lower than youths with attention-deficit/hyperactivity disorder but higher than youths with autism spectrum disorder. Finally, Zhou et al. examine relations between childhood emotional abuse and adult depressive symptoms finding a significant association that is mediated by neuroticism, worsening the prognosis of depression, and by the use of social support and active coping style, playing a protective role for depression. This tentative model for the etiology of depression in adulthood should be verified with a large-scale prospective study.

## Conclusions

This Research Topic focuses on new research perspectives, methodologies, and protocols to understand trajectories and promote change in typical, at-risk, and atypical development. The interplay between early skills and the environment and the connections between theoretical and intervention models with an interdisciplinary, cross-domain and cross-population approach are addressed. These findings have important implications for clinicians and practitioners, who should take into account the specific characteristics of individual and interacting learning processes in planning interventions.

## Author Contributions

AS wrote the first draft of this editorial. KL, AG, ML, MB, and JMI edited consecutive versions. The overall Research Topic has been conceptualized by AS, KL, AG, ML, MB, and JMI. All authors have agreed on the final version.

## Conflict of Interest

The authors declare that the research was conducted in the absence of any commercial or financial relationships that could be construed as a potential conflict of interest.
